# Biology, Ecology and Management of Aquatic Macrophytes and Algae (Volume I)

**DOI:** 10.3390/biology14030246

**Published:** 2025-02-28

**Authors:** Jinlin Liu, Wei Liu, Shuang Zhao

**Affiliations:** 1 Fujian Provincial Key Laboratory of Coastal Basin Environment, Fujian Polytechnic Normal University, Fuqing 350300, China; jlliu@tongji.edu.cn; 2 Project Management Office of China National Scientific Seafloor Observatory, Tongji University, Shanghai 200092, China; 3 State Key Laboratory of Marine Geology, Tongji University, Shanghai 200092, China; 4 College of Environmental and Chemical Engineering, Shanghai University, Shanghai 200444, China

## 1. Introduction

Aquatic macrophytes and algae constitute essential components of aquatic ecosystems, fulfilling diverse and critical roles in sustaining ecological integrity and equilibrium [1,2,3]. Among their most significant ecological functions is their capacity to improve water quality [4]. Through the process of photosynthesis, these organisms assimilate excess nutrients, particularly nitrogen and phosphorus, which are recognized as primary drivers of water pollution. By effectively reducing nutrient concentrations, they play a pivotal role in mitigating eutrophication—a phenomenon that can lead to hypoxic conditions and the formation of dead zones in aquatic systems [5,6]. Moreover, as demonstrated by macrophytes and macroalgae, these organisms exhibit inhibitory effects on the proliferation of microalgal blooms [7,8,9], which are known to pose substantial threats to aquatic biodiversity and human health [10].

In addition to their critical function in water quality regulation, aquatic macrophytes and algae play a vital role as habitats and foraging resources for a diverse array of aquatic fauna [11,12,13]. Their intricate underwater structures provide refuge for fish, invertebrates, and other aquatic organisms, offering protection from predation and establishing optimal conditions for reproduction [14,15,16]. This ecological function, in turn, sustains complex trophic networks, ranging from microscopic plankton to higher trophic-level fish species [4]. Furthermore, the presence of these plants and algae contributes significantly to ecosystem stability by fostering biodiversity.

Given these multifunctional benefits, aquatic macrophytes and algae have become valuable tools in ecological restoration projects. They are extensively employed in the rehabilitation of intertidal zones, lacustrine systems, and riverine ecosystems, where they play a fundamental role in restoring natural habitats and enhancing water quality [17,18,19]. For instance, in intertidal zones, macrophytes contribute to sediment stabilization, mitigate coastal erosion, and promote the recovery of marine biodiversity [20]. Similarly, in freshwater systems, macrophytes improve water clarity and support the proliferation of native species, thereby maintaining ecological balance [21,22].

However, it is crucial to emphasize that the introduction of these macrophytes and algae must be carefully managed. Non-native species, if not properly controlled, can become invasive and disrupt local ecosystems [23,24]. Therefore, ecological restoration projects involving aquatic macrophytes and algae require meticulous planning, continuous monitoring, and adaptive management strategies to ensure their success. When implemented appropriately, these organisms serve as powerful tools in combating environmental degradation.

Meanwhile, in natural ecosystems, certain aquatic macrophytes and algae can become dominant species, often leading to large-scale ecological disasters. For instance, green tides and red tides are two prominent examples [25,26]. These phenomena not only disrupt the ecological balance, but also pose significant challenges to the management and sustainable development of regional ecosystems [27]. The excessive proliferation of these dominant species can result in oxygen depletion, block sunlight penetration, and adversely impact other marine organisms [28]. Consequently, the overall health and biodiversity of the affected ecosystems are severely compromised. These specific ecological disasters primarily include the following:

Firstly, the outbreak of green tides in coastal waters is becoming increasingly severe [29,30], with the genus *Ulva* spp. being the main disaster-causing species. The Southern Yellow Sea of China has become the largest green tide disaster area in the world due to the sharp increase in land-derived nutrient inputs and the suitable hydrodynamic conditions. Meanwhile, areas with severe eutrophication, such as harbors [31], aquaculture zones [32], and intertidal zones with relatively simple habitats [33], are also prone to small-scale green tide disasters.

Secondly, in recent years, the occurrence of golden tides in global oceans has shown a significant increasing trend, with the large floating brown algae *Sargassum* spp. being the main disaster-causing species [34]. Currently, golden tides are primarily observed in the Atlantic and Pacific Oceans (e.g., the Gulf of Mexico, Caribbean Sea, Yellow Sea, East China Sea, and other localized areas), affecting the ecological security of coastal waters in countries such as the United States, Mexico, China, and South Korea [35,36].

Thirdly, red tides, as a global ecological disaster frequently occurring in eutrophic coastal waters, have attracted widespread attention from the academic community due to their increasing frequency, spatial scale, and ecological hazards. Red tides frequently occur in coastal areas near industrial or densely populated zones, as well as in estuaries, in countries such as the United States, Japan, Canada, and China [37,38,39,40]. Red tides not only disrupt the marine ecological balance, causing the death of organisms, but also pose potential threats to human health through the food chain, becoming an urgent global environmental issue that requires transregional collaborative management.

Fourthly, the frequent outbreak of algal blooms (such as cyanobacterial blooms) in lakes, rivers, and brackish water mixing zones worldwide has become a significant challenge for global aquatic ecosystem management [41,42,43]. For example, nearly 50% of large lakes globally have experienced algal bloom events, with European lakes having the highest frequency and severity of algal blooms, followed by North America. In addition, lakes in Central Asia, East Asia, southeastern South America, and eastern and southern Africa also commonly experience algal bloom outbreaks [44,45].

Fifthly, due to poor management, aquatic macrophytes in inland water bodies may also experience small-scale ecological outbreaks. Invasive species with strong reproductive capabilities, such as *Eichhornia crassipes* [46], can rapidly cover the water surface in eutrophic waters, often leading to secondary ecological risks such as river blockages and water quality deterioration, and significantly reducing the flood discharge efficiency of flood control projects. It is worth noting that even *Hydrilla verticillata*, which is commonly used in ecological restoration projects, can accumulate excessive biomass and form “underwater meadows” in eutrophic waters, trapping fishing boats and obstructing water flow, making cleanup difficult.

As demonstrated above, aquatic macrophytes and algae play a dual role in ecosystems. On the one hand, they play an important role in maintaining ecosystem stability and biodiversity. On the other hand, some species may trigger ecological disasters, posing threats to the healthy development of regional ecosystems. Therefore, it is highly urgent for researchers to conduct timely studies on aquatic macrophytes and algae. As three researchers who have long been engaged in the field of ecology, we are well aware that a single article often represents only a very limited range of knowledge points. Only through the format of a Special Issue can we further promote integration and interaction among different sub-disciplines. From a long-term perspective, the establishment of a Special Issue is conducive to the development of the discipline, and thus we have always had the vision of creating a Special Issue.

Fortunately, upon invitation from the editorial department of Biology, we explored the possibility of creating this Special Issue and ultimately determined the relevant details of its establishment, with the aim of providing an academically inclusive platform for scholars in the relevant research fields to publish their academic achievements and promote open discussions. Initially, this Special Issue was named “Biology, Ecology, and Management of Aquatic Macrophytes”. With the development of modern taxonomy, scientists have come to recognize that some algae (e.g., *Saccharina japonica*) do not belong to the plant kingdom [47], but rather to the protist kingdom, and thus the term “algae” is more accurate than “macrophytes”. Therefore, the name of this Special Issue was ultimately changed to “Biology, Ecology, and Management of Aquatic Macrophytes and Algae”. Although the modern taxonomic system is in a continuous state of dynamic change with the development of molecular biological classification techniques [48,49], the name of this Special Issue, determined based on the current taxonomic system, is accurate.

Meanwhile, it is also crucial to determine the research topics for this Special Issue. There are hundreds of research directions related to the theme of “Biology, Ecology, and Management of Aquatic Macrophytes and Algae”. Combining our respective strengths in our research fields, the three Guest Editors have decided to focus on the following five main research topics: (1) the key role of aquatic macrophytes and algae in ecosystems; (2) the application of aquatic macrophytes and algae in ecological restoration engineering; (3) the allelopathy of aquatic macrophytes and algae toward algal bloom species (red tide and cyanobacterial bloom); (4) ecological risks and management measures associated with macroalgal blooms or invasive species; and (5) biodiversity conservation and ecological management issues to be considered in the process of ecological restoration. Whether our Special Issue will receive submissions on these topics depends partly on serendipity and a bit of luck.

Luckily, within half a year, we received approximately 20 manuscript submissions or pre-reviews, which far exceeded our expectations and by which we are truly honored. After undergoing a rigorous multi-round anonymous peer-review process conducted by the editorial department, 10 articles were ultimately accepted for publication. We extend our congratulations to all the authors of these 10 papers. Meanwhile, for those whose manuscripts were unfortunately rejected, we also believe that your submissions are academically significant. Perhaps you lacked some luck during the peer-review stage. We look forward to your continued support of our Special Issue in the future.

## 2. An Overview of the Published Articles

The 10 articles ultimately accepted and published in this Special Issue were authored primarily by researchers from three countries: China, Peru, and the United States. A total of 14 research institutions were involved, with 12 from China, 1 from Peru, and 1 from the United States. These institutions are all recognized as famous scientific research organizations globally.

**Figure 1:**
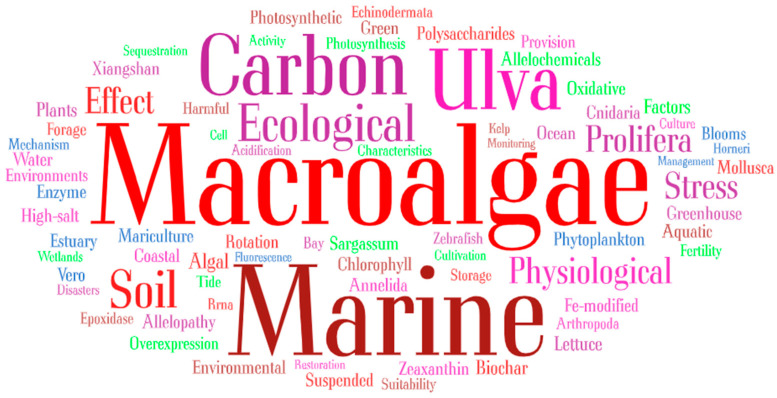
Word cloud of the 10 articles in the Special Issue.

This Special Issue mainly focuses on the ecology of macrophytes and algae, including various living environments of macrophytes and algae, systematically exploring their interactions with diverse habitats (marine, coastal soils, and climate-vulnerable ecosystems) through interdisciplinary lenses encompassing biology, systematics, environmental science, and aquaculture management. Figure 1 highlights the foci of this Special Issue: macroalgae and the marine environment, particularly *Ulva prolifera.* For the sake of argument, we have divided the 10 articles into 5 parts: macrophytes, macroalgae, microalgae, ecological management of hazardous macroalgae, and ecological disasters and disaster prevention/mitigation.

### 2.1. Macrophytes

Macrophytes play indispensable roles in global ecosystems by sustaining biodiversity, maintaining ecological equilibrium, and promoting human well-being. They deliver critical ecosystem services, including water purification, carbon sequestration, habitat provision for aquatic organisms, and flood regulation through the root stabilization of sediments. These services are vital for both environmental health and sustainable human development.

Macrophytes exhibit sophisticated stress-resistance mechanisms when confronting environmental adversities such as salinity, heat stress, drought, and low-temperature stress, endowing them with remarkable adaptive plasticity [50]. However, this formidable adaptive capacity, compounded by globalization, has facilitated the global proliferation of invasive macrophyte species. Excessive colonization by invasive species can obstruct waterways, block sunlight, cause oxygen deprivation in the water, and directly threaten native biodiversity. Counterintuitively, Zamora-Aranda and Aponte [51] reevaluated the invasive macrophyte *Pistia stratiotes* in Peru’s Santa Rosa Wetland through an ecosystem service lens. Employing field measurements and spatial modeling, they quantified the wetland’s *P. stratiotes* biomass at 37,809.99 metric tons, equivalent to 1075.25 tons of sequestered carbon, 3942.57 tons of CO₂ mitigation, 2134.41 tons of forage potential, and 339.05 tons of protein storage. This finding underscores *P. stratiotes*’ dual service potential in carbon capture and nutritional provisioning, demonstrating how strategically managed macrophytes can function as “low-cost ecological engineers” even in arid coastal zones with water scarcity.

However, we must also recognize that the management of macrophytes requires a cautious approach. Some macrophytes have strong reproductive capabilities, which can lead to the formation of dominant populations and even result in them becoming invasive species that threaten local ecosystems. Therefore, when utilizing the ecosystem services provided by macrophytes, it is essential to fully consider their potential risks and implement scientifically sound management measures to achieve a balance between ecological and economic benefits.

### 2.2. Multidimensional Research on Macroalgae Cultivation and Marine Environmental Changes

In recent years, the intensification of global marine environmental changes, coupled with the rapid expansion of the macroalgae cultivation industry, has underscored the need to optimize cultivation practices, enhance ecological and economic value, and address the challenges posed by shifting environmental conditions. These priorities have emerged as critical focal points in marine scientific research. In this Special Issue of Biology, four studies explore the key dimensions of macroalgae cultivation, including rotational cultivation models, the influence of environmental factors on growth dynamics, and the potential applications of macroalgae-derived polysaccharides in biomedicine. Collectively, these investigations provide robust theoretical foundations and practical insights to advance the sustainable development of macroalgae cultivation.

#### 2.2.1. Research on Rotational Cultivation Models of Macroalgae Based on Photosynthetic Physiological Characteristics

Currently, global macroalgae cultivation is predominantly characterized by monoculture practices, which face challenges such as a lack of industrial diversity and limited resilience to risks. To address these issues, Chen et al. [52] conducted a study focusing on the rotational cultivation of three macroalgae species—*Hizikia fusiformis*, *S. japonica*, and *Gracilariopsis lemaneiformis*—in Xiangshan Bay, Zhejiang Province. The study involved in situ experiments to monitor these species’ photosynthetic activity responses under varying temperature and light conditions. The results revealed significant differences in the tolerance of the three species to light and temperature: the effective quantum yield of photosynthesis in *H. fusiformis* and *G. lemaneiformis* remained stable under fluctuating light conditions, while that of *S. japonica* initially decreased before recovering. In terms of temperature, *H. fusiformis* and *S. japonica* exhibited their highest relative electron transport rates in May (20.3 °C), whereas *G. lemaneiformis* showed optimal growth in September (27.5 °C).

Based on these findings, Chen et al. [52] developed a rotational cultivation model for macroalgae, tailored to the seasonal temperature variations in the coastal waters of the Shandong, Zhejiang, and Fujian provinces in China. This model selects suitable macroalgae species according to specific temperature conditions, aiming to achieve stable year-round production and provide theoretical support for the construction of artificial macroalgae fields in marine ranching systems. Such an approach enhances both ecological and economic benefits. Chen et al. [52] offer innovative insights into overcoming the limitations of monoculture practices, highlighting the importance of rotational cultivation models based on ecological and physiological characteristics for improving the sustainability of macroalgae cultivation. However, the research primarily focused on the effects of temperature and light on photosynthetic physiology. Future studies could further investigate the integrated impacts of additional environmental factors, such as nutrient availability and water flow, on macroalgae growth.

#### 2.2.2. Impact of Environmental Changes on *Saccharina japonica* Cultivation

In practical applications of macroalgae cultivation, changes in environmental factors significantly influence cultivation outcomes. Bao and Xu [53] conducted an in-depth study on the effects of environmental changes on *S. japonica* cultivation in Xiangshan Bay. The research, conducted from November 2020 to May 2021, involved five observational cruises to systematically analyze the variations in physicochemical environmental factors, including temperature, salinity, light, nutrient concentrations, and ocean currents, and their impacts on the growth suitability of *S. japonica*.

The results revealed that temperature is the decisive factor influencing *S. japonica* cultivation in Xiangshan Bay. The suitability index for temperature ranged from 0.02 to 0.94 during the observation period, indicating that temperature fluctuations had the most significant impact on growth. In contrast, the suitability indices for salinity and light conditions ranged from 0.96 to 0.99 and 0.97 to 1.00, respectively, demonstrating that the salinity and light conditions in Xiangshan Bay are highly favorable for *S. japonica* cultivation, and do not limit the expansion of the cultivation time window. Additionally, nutrient concentrations remained at high suitability levels during the later stages of cultivation (DIN: 0.96–0.97; DIP: 0.92–0.95), suggesting that the current scale of *S. japonica* cultivation in Xiangshan Bay has not yet reached the carrying capacity of the area, leaving room for further development. Although *S. japonica* cultivation led to a noticeable reduction in surface current intensity, it did not significantly affect nutrient replenishment, indicating that cultivation has not yet exhibited a pronounced self-limiting effect in Xiangshan Bay.

In summary, temperature is the key limiting factor for *S. japonica* cultivation in Xiangshan Bay, while salinity and light conditions are highly conducive to its growth. Future research should focus on the risks posed by temperature fluctuations to *S. japonica* cultivation and explore the breeding of heat-resistant varieties to further enhance the efficiency and sustainability of cultivation in Xiangshan Bay.

#### 2.2.3. Coupled Effects of Ocean Acidification and Temperature on Macroalgae

In the context of global climate change, ocean acidification and rising temperatures pose dual threats to marine ecosystems. Wang et al. [54] focused on this issue, using *Ulva fasciata* and *Sargassum horneri* collected from Ma’an Archipelago as experimental subjects. Through a series of cultivation experiments under different temperatures (15 °C and 20 °C) and carbon dioxide concentration (400 μL/L and 1000 μL/L) conditions, they investigated the interactive effects of ocean acidification and temperature changes on the photosynthetic physiology of these two macroalgae. Key indicators such as rapid light curves, fluorescence induction parameters, relative growth rates, dissolved organic carbon release, and photosynthetic pigment content were measured to comprehensively assess the responses of the macroalgae.

The study found that at 20 °C, ocean acidification reduced the tolerance of *U. fasciata* to high light intensity, exacerbating photoinhibition. However, at 15 °C, while temperature significantly inhibited the growth and photosynthetic activity of *U. fasciata*, ocean acidification alleviated this inhibition and promoted its growth. For *S. horneri*, ocean acidification similarly reduced its tolerance to high light intensity at 20 °C, but this inhibitory effect was less pronounced at 15 °C, where ocean acidification further enhanced its growth. Additionally, the relative growth rate, photosynthetic pigment content, and dissolved organic carbon release of *U. fasciata* were primarily influenced by temperature, with 20 °C being more conducive to its growth and metabolite synthesis. In contrast, *S. horneri* exhibited faster growth and higher metabolic activity at 15 °C.

Overall, ocean acidification reduces the tolerance of macroalgae to high light intensity under optimal growth temperatures, while under suboptimal temperatures, it mitigates the inhibitory effects of temperature and promotes growth. This study provides valuable insights into how macroalgae may respond to future oceanic climate changes in natural environments. Future research should focus on the long-term adaptive changes in macroalgae under the coupled effects of ocean acidification and temperature, and explore the cultivation of macroalgae varieties adapted to changing marine environments, thereby enhancing the sustainability of marine ecosystems.

#### 2.2.4. Antioxidant Activity and Application Potential of *Sargassum horneri* Polysaccharides

In addition to the ecological and environmental effects of seaweed cultivation, the bioactive substances derived from seaweeds also hold significant developmental value. The study by Wei et al. [55] focused on the antioxidant activity of *S. horneri* polysaccharides (SHP). With increasing environmental complexity, excessive production of reactive oxygen species (ROS) in the human body can lead to oxidative stress, thereby harming health. Given that polysaccharides extracted from seaweeds possess various biological activities and the proliferation of *S. horneri* poses environmental challenges, the development of SHP resources is of great importance.

The study evaluated the antioxidant activity of SHP by inducing oxidative stress in African green monkey kidney cells (Vero cells) and zebrafish using hydrogen peroxide (H₂O₂). The experimental results demonstrated that SHP exhibited no significant toxicity to Vero cells and showed a protective effect; it significantly increased the viability of treated Vero cells, effectively reduced intracellular ROS levels, enhanced superoxide dismutase (SOD) activity, and decreased malondialdehyde (MDA) content. In zebrafish experiments, SHP also demonstrated protective effects, improving the survival rate of H_2_O_2_-induced zebrafish embryos, reducing embryonic malformations, lowering heart rate, and enhancing the activities of SOD, catalase (CAT), and glutathione peroxidase (GSH-PX), while reducing MDA and ROS levels.

In summary, SHP effectively protected Vero cells and zebrafish from H₂O₂-induced oxidative damage, demonstrating its potential as a natural antioxidant. This study provides new insights into the mechanisms of antioxidant activity in biological systems and offers a novel approach to mitigating the environmental pressure caused by *S. horneri* and realizing its resource utilization. Future research should focus on the long-term antioxidant effects of SHP in different biological systems and complex environments, and actively explore its potential for application in industries such as biomedicine, cosmetics, and food, thereby further enhancing its positive impacts on ecosystems and human health.

### 2.3. Microalgae

Microalgae, as the most abundant biological group in aquatic ecosystems, serve as primary producers and play crucial roles in material cycling and transformation, energy flow, and ecological regulation [56]. Their high sensitivity to environmental changes makes them effective bioindicators. Intensive human activities, particularly rapid urbanization and population aggregation in coastal bay areas, frequently lead to environmental challenges such as water eutrophication and red tide outbreaks. Therefore, a comprehensive understanding of the diversity distribution, spatial heterogeneity patterns, and environmental gradient responses of freshwater, estuarine, and marine microalgae communities holds immeasurable value for pollution source tracking, water quality assessment, and ecosystem health evaluation.

In China’s highly urbanized Pearl River Estuary, Xia et al. [57] employed in situ fluorescence parameter measurements coupled with 18S rRNA sequencing to reveal that Chlorophyta and Cryptophyta dominate freshwater areas, while Dinophyta and Haptophyta prevail in marine zones. The algal distribution demonstrates significant correlations with temperature and salinity gradients. Notably, nutrient levels (NO_3_^−^-N, PO_4_^3−^-P, and SiO_3_^2−^-Si) show marked associations with the Simpson index, suggesting a linear relationship between eutrophication intensity and microalgae community structure. These findings provide valuable references for water management strategies in this region.

In conclusion, complex interactions exist between microalgae community structure and environmental factors in the Pearl River Estuary. Future research should focus on three key directions: (1) elucidating the response mechanisms of specific algal taxa to particular pollutants to establish refined bioindicator systems; (2) integrating long-term monitoring data to predict climate change and the anthropogenic impacts on microalgae communities, informing adaptive management strategies; and (3) applying metagenomics and other advanced techniques to decode the functional gene composition of microalgae communities, thereby comprehensively assessing their roles in material cycling and energy flow. Such multidisciplinary approaches will enhance our understanding of estuarine ecosystem dynamics.

### 2.4. Ecological Management of Hazardous Macroalgae

#### 2.4.1. Biological Characteristics of the Harmful Macroalga *Ulva prolifera*

A prominent focus of this Special Issue is the recurrent discussion of *U. prolifera*, a disaster-causing macroalga. Under favorable environmental conditions, *U. prolifera* can rapidly proliferate and form massive floating aggregates, a phenomenon termed “green tides”. While water eutrophication is recognized as a key trigger for green tide outbreaks, the genetic traits of *U. prolifera* itself have emerged as a critical research frontier in green tide biology. As a euryhaline species, *U. prolifera* thrives in environments with drastic salinity fluctuations, such as intertidal zones and estuaries. Its salinity tolerance mechanisms have long attracted scientific attention. He et al. [58] recently elucidated the molecular basis of its adaptation to high salt stress. Their study cloned and analyzed the zeaxanthin epoxidase (ZEP) gene (UpZEP) in *U. prolifera*, revealing that high salinity significantly enhances xanthophyll accumulation and induces UpZEP expression. This gene plays a central role in protecting the photosynthetic apparatus from photodamage under abiotic stress by regulating xanthophylls synthesis, thereby conferring a competitive advantage over other algae in high-salinity environments. This discovery offers novel insights into the mechanisms driving green tide formation.

While He et al. [58] uncovered the genetic foundations of *U. prolifera*’s salt adaptation, Zeng et al. [59] further explored its ecological competitiveness through allelopathy. In their review, Zeng et al. [59] systematically cataloged allelochemicals produced by *U. prolifera*, including fatty acids, aldehydes, phenols, and terpenoids. These compounds exert broad environmental impacts, not only inhibiting the growth of competing algae, but also influencing marine microbial communities, mycorrhizal fungi, and invertebrates, thereby reshaping ecosystems. The development of rapid detection technologies for these allelochemicals could enhance the real-time monitoring of green tide dynamics, unravel complex interactions between algal blooms and their environments, and advance early warning systems for coastal ecological disasters.

Notably, some allelochemicals highlighted in this review demonstrate dual regulatory effects on green tides, red tides, and the growth of invasive species like *Spartina alterniflora*. This underscores the inseparable interplay between marine and terrestrial ecosystems in coastal zones. Consequently, mitigating marine ecological disasters requires integrated land–sea management strategies. Reducing pollutant and nutrient discharges into coastal waters, coupled with enhancing carbon, nitrogen, and phosphorus sequestration in terrestrial soils, could alleviate eutrophication pressures and curb the frequency and intensity of algal blooms. Such interdisciplinary approaches are essential for balancing ecological health with sustainable coastal development.

#### 2.4.2. Disaster Prevention and Control from a Land–Sea Integrated Perspective

The frequent occurrence of green tides poses severe threats to coastal economies and ecological security. These events are not isolated marine environmental issues, but rather manifestations of systemic imbalances between terrestrial and marine ecosystems. Current mitigation strategies predominantly focus on offshore interception (e.g., the removal of nori aquaculture zones in Jiangsu Province) or end-of-pipe pollution control. However, Niu et al. [60] propose an innovative approach: enhancing the carbon sequestration capacity of urban green spaces to improve terrestrial nutrient retention and purification, thereby reducing the flux of nitrogen, phosphorus, and other pollutants into marine environments and indirectly alleviating coastal eutrophication.

The study explores the effects of Fe-modified biochar combined with plant growth-promoting bacteria (PGPB) on the fertility and carbon retention of urban green space soils. The results indicate that, compared to the application of biochar or PGPB alone, the combination of iron-modified biochar and PGPB significantly reduces soil pH, mitigates soil alkalization, increases the content of alkaline nitrogen in the soil, and improves soil aggregate stability. These changes enhance soil fertility and ecological function. Furthermore, this treatment significantly increases the levels of soil organic carbon (SOC), particulate organic carbon (POC), and soil inorganic carbon (SIC), thereby boosting the soil’s carbon retention capacity. This approach holds promise for reducing the entry of nutrients such as carbon, nitrogen, and phosphorus into the ocean.

In summary, adopting a land–sea integrated perspective for disaster prevention and control is essential. By enhancing the ecological functions of terrestrial systems, we can create a more balanced land–sea interaction, ultimately contributing to the mitigation of marine ecological disasters such as green tides. This holistic approach not only addresses the symptoms of the problem, but also targets the underlying causes, paving the way for sustainable coastal management and ecological resilience.

### 2.5. Ecological Disasters and Disaster Prevention/Mitigation

Yao et al. [61] conducted a comprehensive review of the current status of marine ecological disasters in China, with a focused analysis on disasters triggered by marine organisms. Their study systematically examined the impacts of these disasters on marine ecosystems, fisheries, tourism, and coastal industries. Yao et al. [61] emphasized that global climate change and intensified human activities have disrupted the marine ecosystem equilibrium, leading to frequent occurrences of marine biological disasters. Beyond commonly observed green tides, golden tides, and red tide outbreaks, proliferations of marine organisms such as Cnidaria (jellyfish), Annelida (*Urechis unicinctus*), Mollusca (*Philine kinglipini*), Arthropoda (*Acetes chinensis*), and Echinodermata (*Acaudina molpadioides*, Asteroidea, and Ophiuroidea) have significantly impacted the sustainability of marine ecological resources and human socio-economic activities. These biological outbreaks not only destabilize marine ecosystem integrity and biodiversity, but also pose substantial threats to the safety of coastal nuclear power plants, recreational zones, and nearshore infrastructure.

Yao et al. [61] provided a detailed analysis of the environmental drivers, causative mechanisms, and ecological consequences of these marine organism outbreaks. To address these challenges, Yao et al. [61] proposed strategic recommendations, including the following: enhanced monitoring and early warning systems for marine ecological disasters, with expanded coverage of biological threats; strengthened resource utilization strategies; and foundational research initiatives. These measures aim to improve preparedness for future marine biological disasters while safeguarding the health and sustainable development of marine ecosystems.

## 3. Summary and Outlook

We are pleased to report that the Special Issue entitled “Biology, Ecology, and Management of Aquatic Macrophytes and Algae” has achieved significant progress. A total of 10 articles were accepted and published, garnering widespread recognition from the research community. Notably, this Special Issue has begun to establish its academic influence within the field, which is particularly encouraging for the editorial team.

While the Special Issue encompasses both aquatic macrophytes and algae, the submitted content did not fully align with our initial expectations. Notably, manuscripts focusing on aquatic macrophytes were underrepresented, with algae-related studies dominating the issue. Furthermore, research on marine environments prevailed, while studies on freshwater, estuarine, and other habitat types were limited. We attribute this imbalance to several factors: (1) the disciplinary backgrounds of the Guest Editors, which are centered on marine ecosystems and algae, likely influenced the thematic focus; (2) insufficient promotion of the Special Issue within the aquatic macrophyte research community; and (3) lingering reservations among some scholars about publishing in OA journals. Despite these challenges, we persevered and successfully completed this scholarly work. Looking ahead, we are excited about the direction of submissions for the next Special Issue (Volume II). We believe that the next issue can focus more on the following four research themes:

① Advancing Research on Macrophytes in Freshwater and Coastal Environments

Future studies should emphasize the ecological roles of macrophytes in freshwater and coastal ecosystems, particularly their responses to environmental changes and applications in ecological restoration. Examples include submerged macrophytes’ remediation potential in eutrophic waters or emergent macrophytes’ habitat functions in wetland ecosystems. Submissions may address ecological functions, community dynamics, climate change responses, or optimization of restoration strategies, though other relevant topics are also welcome.

② Expanding Research on Allelopathy and Biodiversity Conservation

We encourage studies on allelopathic interactions between aquatic macrophytes/algae and harmful algal bloom species, with emphasis on the mechanisms and applications for bloom suppression. Concurrently, research on balancing biodiversity conservation with ecosystem functionality during restoration processes should be prioritized. Potential topics include the identification of allelochemicals, biodiversity protection strategies, and sustainable management in restoration contexts.

③ Promoting Integrated Land–Sea Management Approaches

Given the interconnectedness of terrestrial and marine ecosystems, future research should adopt a holistic perspective to improve aquatic ecosystem health through the systemic management of agricultural and urban runoff. Submissions may explore the comprehensive impacts of land-based pollution, integrated management frameworks, or cross-regional restoration practices.

④ Enhancing Long-Term Monitoring and Adaptive Management

The long-term monitoring of aquatic macrophytes and algae is critical for understanding their responses to environmental shifts and developing adaptive management strategies. The incorporation of advanced technologies (e.g., remote sensing, molecular biology, or metagenomics) to track community structure and functional changes is strongly encouraged. Topics may include the establishment of ecological monitoring networks, adaptive management frameworks, or assessments of ecosystem services.

Finally, we extend our deepest gratitude to all authors, anonymous reviewers, assistant editors (especially Joey Li for his exceptional support), and academic editors involved in this publication. We regret that some scholars could not submit manuscripts within the tight timeframe and look forward to their contributions in the next Special Issue (Volume II). See you then.
